# Beyond Survival: Understanding Ethnic and Socioeconomic Disparities in Post-Cancer Healthcare Use in England

**DOI:** 10.3390/cancers18010047

**Published:** 2025-12-23

**Authors:** Tahania Ahmad, Abu Z. M. Dayem Ullah, Claude Chelala, Stephanie J. C. Taylor

**Affiliations:** 1 Wolfson Institute of Population Health, Queen Mary University of London, London E1 2AB, UK; s.j.c.taylor@qmul.ac.uk; 2 Barts Cancer Institute, Queen Mary University of London, London EC1M 6AU, UK; d.ullah@qmul.ac.uk (A.Z.M.D.U.); c.chelala@qmul.ac.uk (C.C.)

**Keywords:** cancer survivors, healthcare utilisation, living with and beyond cancer, ethnicity, socioeconomic status, primary care consultations, hospital admissions

## Abstract

**Background:** Cancer survivors represent a growing proportion of the UK population and often experience higher multimorbidity and healthcare needs. However, limited research in the UK has explored ethnic and socioeconomic disparities in healthcare resource use among long-term cancer survivors. **Methods:** Using linked primary care (Clinical Practice Research Data) and secondary care (Hospital Episode Statistics–Admitted Patient Care) data between 2010 and 2020, this population-based cohort study compared healthcare utilisation among 170,352 cancer survivors and 415,975 matched controls without a cancer diagnosis. Outcomes included primary care consultations and hospital admissions (planned and emergency). Analyses adjusted for age, sex, body mass index, smoking, ethnicity, and the Index of Multiple Deprivation. Negative binomial models were used to estimate incidence rate ratios (IRRs). **Results:** Cancer survivors averaged 33 more primary-care consultations over ten years than controls, with Pakistani, Indian, and White survivors recording the higher rates. Hospital admissions were consistently higher among survivors across all age groups, peaking in those aged 60–75 years. Planned admissions were highest among Black Caribbean (IRR 1.80 (95% CI 1.73–1.87)), Pakistani (IRR 1.71 (1.63–1.78)), and Bangladeshi (IRR 1.66 (1.53–1.80)) groups. Emergency admissions followed a similar trend, remaining statistically significant only for Pakistani survivors (IRR 1.23 (1.16–1.30)). A strong socioeconomic gradient was observed, with healthcare utilisation increasing as deprivation worsened. **Conclusions:** Cancer survivors experience substantially greater healthcare use than matched controls, with persistent ethnic and socioeconomic disparities. Strategies to reduce disparities should focus on earlier diagnosis, enhanced long-term care coordination, and culturally informed interventions addressing both cancer survivorship and multimorbidity.

## Simple Summary

Cancer survivors are living longer, but many continue to need more medical care than people who have never had cancer. This study looked at healthcare use among over half a million adults in the UK to understand how cancer affects long-term care needs across different ethnic and socioeconomic groups. By linking primary care and hospital data, the researchers found that cancer survivors had many more GP visits and hospital admissions than similar people without cancer. These differences were especially large for Pakistani, Bangladeshi, and Black Caribbean survivors, and for those living in more deprived areas. The findings suggest that even within the NHS, disparities in post-cancer care persist. Improving early diagnosis, care coordination, and culturally sensitive support could help ensure that all cancer survivors receive the long-term care they need.

## 1. Introduction

Cancer survivorship has increased markedly over the past two decades due to improvements in detection and treatment, resulting in a growing population with potentially complex healthcare needs. Cancer survivors in England experience elevated risks of comorbidities and multimorbidity [1], contributing to greater reliance on healthcare services. Previous research found that cancer survivors from ethnic minorities present at a later stage of cancer [2,3] and encounter higher rates of emergency cancer admissions, either due to emergency presentations during diagnoses or because of poorer outcomes during palliative care [4]. Despite the universal coverage of the National Health Service (NHS), substantial disparities persist across ethnic and socioeconomic groups in the UK, influencing access, utilisation, and outcomes of care. A sensible way to measure this is through healthcare resource use.

Previous UK studies have shown that long-term cancer survivors, particularly those with breast, colorectal, and prostate cancers, have significantly higher primary-care consultation rates than individuals without cancer, with these rates gradually declining over time [5]. However, research since 2011 on healthcare utilisation patterns among UK cancer survivors remains scarce. Similar phenomena of increased consultations have been observed in France and Norway [6,7]. A Swedish case–control study found that the frequency of consultation and the number of symptoms and diseases expressed in diagnostic codes rose in tandem 50–100 days before the cancer diagnosis [8] while a Danish study found an increased rate of consultations before a diagnosis of cancer recurrence or a second primary cancer [9].

In the UK, minority ethnic groups often present with cancer at later stages, experience poorer outcomes, and have higher rates of emergency presentation compared to the white population [4]. Socioeconomic deprivation also correlates with higher cancer mortality and lower screening uptake [10,11]. Here, we aim to quantify differences in primary and secondary care use between cancer survivors and matched controls, assess ethnic variation, and examine the influence of socioeconomic status. Understanding these differences is critical for developing targeted interventions to promote equitable survivorship care across diverse populations and to allocate NHS funding where it is most needed.

## 2. Methods

### 2.1. Study Design and Data Sources

A retrospective cohort study design was employed using linked data from the Clinical Practice Research (CPRD), Hospital Episode Statistics–Admitted Patient Care (HES-APC) and the Index of Multiple Deprivation (IMD). CPRD provides anonymised primary-care data [12].

### 2.2. Population and Follow-Up

The study included adults (≥18 years) diagnosed with any from a list of 28 cancer sites between 1 January 2010 and 31 December 2018, allowing for at least two years of post-diagnosis follow-up. Exposure was defined as a recorded cancer diagnosis of one of the 28 cancers outlined in the MULTIPLY initiative code lists [13]. These 28 cancers were not comprehensively explored in previous studies. The earliest recorded diagnosis date in CPRD was taken as the index date. Only individuals alive two years after cancer diagnosis were considered survivors, ensuring exclusion of palliative cancer cases and acute-phase treatment effects. Cancer survivors were matched 1:3 to controls without cancer by age, sex, and general practice. The follow-up period extended until the earliest event of death, record termination, or 31 December 2020.

### 2.3. Outcomes

Three primary outcomes measured between 2010 and 2020 were:

- Primary care consultations: Total number and annual rates of any recorded consultation with a general practitioner or other primary care staff.
- Planned hospital admissions: Elective admissions identified within HES-APC.
- Emergency hospital admissions: Unplanned or urgent admissions, excluding maternity-related episodes.

Admissions were defined as continuous hospital stay; transfers between hospitals were counted once at the initial hospital.

### 2.4. Covariates

Covariates included age, sex, body mass index (BMI), smoking status, IMD quintile and ethnicity (12 CPRD categories—White, Indian, Bangladeshi, Pakistani, Chinese, Other Asian, Black Caribbean, Black African, any other Black, Mixed, Others and Unknown) as these were considered potential confounders in the previous literature [14,15,16]. BMI values were averaged across available records to account for fluctuations during and after cancer treatment. Smoking status was classified as never, former, or current. Multimorbidity was quantified as a count of 29 long-term conditions.

### 2.5. Statistical Analysis

Descriptive analyses summarised cohort characteristics. Incidence rates and incidence rate ratios (IRRs) with 95% confidence intervals (CIs) for primary care consultations and hospital admissions were estimated by comparing cancer survivors with matched non-cancer controls. Models were adjusted for age, sex, BMI, smoking, ethnicity, IMD, multimorbidity and cancer site. A complete case analysis was conducted because the substantial sample size ensured adequate statistical power despite missing data.

To better model recurrent admissions, the study explored event-based survival models (e.g., Andersen–Gill extensions of the Cox model), zero inflation model, and count-based regression approaches (Poisson and negative binomial) [17,18]. The Poisson model assumes equal mean and variance, which does not hold for hospital admissions, as some patients experience multiple events while others have none [19]. As the primary care consultation data is over dispersed and does not have similar rates over time, negative binomial models were selected to estimate the incidence rate ratio of primary care consultations (PCC).

Numerous studies have indicated that negative binomial models offer a more accurate model fit for admissions data compared to Poisson models [17,19]. Admission data may exhibit zero inflation, indicating an overrepresentation of zero values. Strategies to address this include employing zero-inflated Poisson or negative binomial models, as well as hurdle models. These two-part models estimate both the probability of non-zero admissions and the count of admissions. They generally demonstrate comparable performance in various situations [18]. To assess model fit, we compared standard and zero-inflated versions of Poisson and negative binomial models, utilising Akaike Information Criteria (AIC), Bayesian Information Criteria (BIC) and Vuong test [20]. The negative binomial with clustered standard errors (SEs) provided the lowest AIC and best fit. Additionally, we incorporated clustered standard errors to address potential clustering by primary care practice.

## 3. Results

### 3.1. Cohort Characteristics

Of 1,151,327 eligible patients, 586,327 met inclusion criteria after restricting to the HES-APC linkage period (2010–2020). This comprised 170,352 cancer survivors and 415,975 matched non-cancer controls. As shown in Table 1, survivors were older (mean ≥60 years), predominantly White (91.7%), and exhibited higher multimorbidity prevalence than controls (95.8% vs. 94.8%). Distributions of BMI, smoking, and deprivation were similar between groups.

### 3.2. Primary-Care Consultations

Cancer survivors recorded consistently higher rates of primary-care consultations across all age groups compared with controls. Consultation frequency increased with age, peaking among those aged 75–90 years. Over the 10-year period, survivors had an average of 172 primary-care consultations compared with 145 in controls.

**Figure 1:**
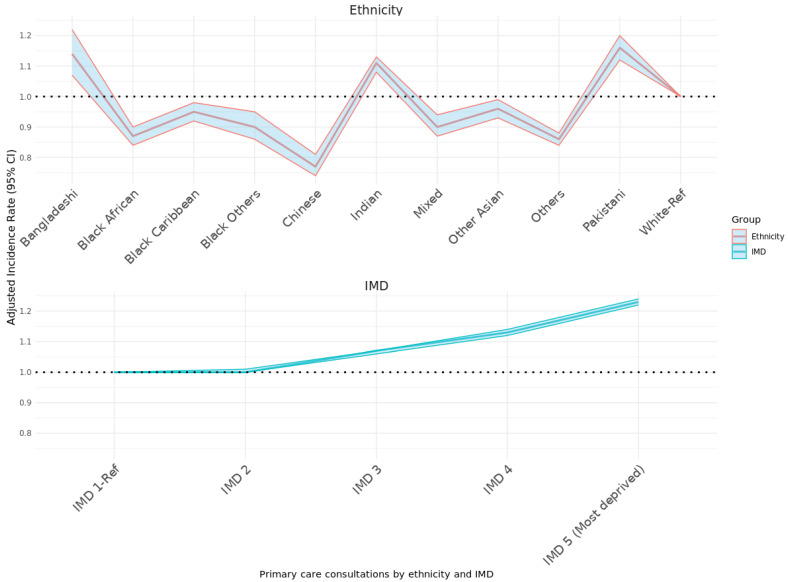
Adjusted incidence rate (95% CI) of primary cancer consultations by ethnicity and IMD.

Ethnic differences were substantial (Table 2). Pakistani (188 primary-care consultations), Indian (182), and White (172) survivors had the highest mean PCC. The lowest counts occurred in the Chinese (128) and “Black Other” (132) groups. Adjusted IRRs confirmed significantly higher consultation rates among Pakistani (IRR 1.16; 95% CI 1.12–1.20) and Bangladeshi (1.14; 95% CI 1.07–1.22) survivors relative to White survivors, whereas PCC was lower amongst all other ethnicities. Chinese survivors had markedly lower rates (0.77; 95% CI 0.74–0.81) (Figure 1).

A clear socioeconomic gradient was also observed (Figure 1). PCC increased with deprivation: survivors in the most deprived IMD quintile averaged 175 consultations per 10 years/over the 10 years? compared to 161 in the least deprived quintile, yielding an adjusted IRR of 1.23 (95% CI 1.22–1.24).

### 3.3. Hospital Admissions

**Figure 2:**
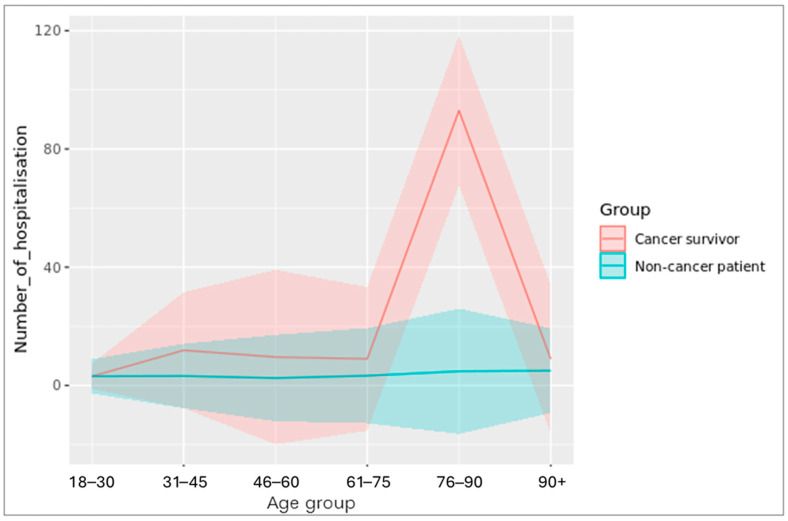
Total number of hospitalisations in survivors compared to matched controls by age group.

Cancer survivors had substantially higher numbers of total hospitalisations than controls across all age groups (Figure 2). Admissions peaked between 75 and 90 years—coinciding with the typical age of cancer diagnosis—and were slightly elevated again among younger survivors (30–45 years), reflecting cancers such as cervical, testicular, and brain tumours [21].

**Figure 3:**
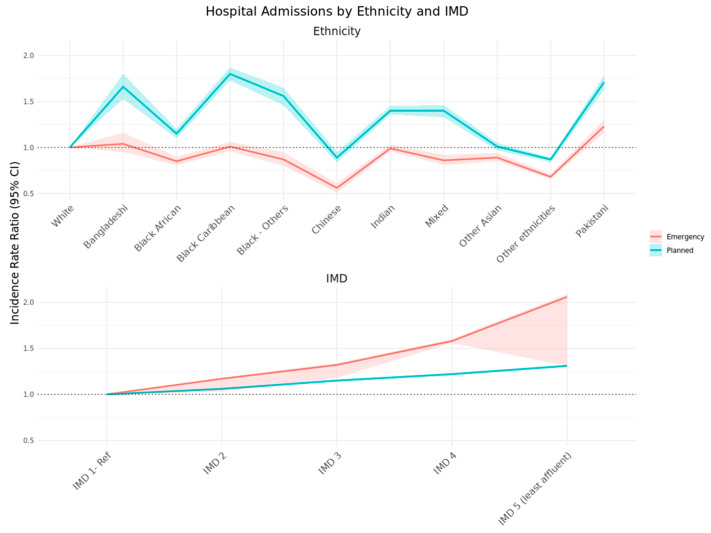
Adjusted planned and emergency admission- incidence rate ratio (95% CI) by ethnicity and IMD.

Ethnic and socioeconomic variations in both planned and emergency admissions were present (Table 3). The highest incidence rates of planned admissions were observed among Black Caribbean (2.72 per 1000 person-years), Pakistani (2.79), and Bangladeshi (2.10) survivors. Adjusted IRRs confirmed significantly higher rates compared to White survivors: Black Caribbean 1.80 (95% CI 1.73–1.87), Pakistani 1.71 (1.63–1.78), and Bangladeshi 1.66 (1.53–1.80) (Figure 3). Planned admission rates also increased with socioeconomic deprivation. Survivors in the most deprived quintile had 31% higher adjusted admission rates than those in the least deprived quintile (IRR 1.31; 95% CI 1.30–1.32) (Figure 3).

In terms of emergency admissions, Pakistani survivors exhibited the highest adjusted incidence (IRR 1.23; 95% CI 1.16–1.30) compared with White survivors (Figure 3). Although Black Caribbean and Bangladeshi groups showed elevated unadjusted rates, these differences were not statistically significant after adjustment. Chinese and “Other” ethnic groups consistently had lower emergency admission rates (IRR 0.56 and 0.68, respectively). A pronounced deprivation gradient persisted: emergency admission rates more than doubled between the most affluent (IMD 1) and most deprived (IMD 5) quintiles (adjusted IRR 2.06; 95% CI 2.03–2.09).

## 4. Discussion

This large, population-based analysis demonstrates that cancer survivors use significantly more primary-care and hospital resources than matched individuals without cancer. It is well established that ethnic minorities have more comorbidities [22,23,24,25] and this pattern extends to cancer survivors [26]. Our study expands on previous UK research by incorporating detailed ethnic and socioeconomic stratifications, revealing persistent disparities in survivorship care despite universal health coverage under the NHS.

Cancer survivors had substantially higher primary-care consultation rates across all age groups, averaging 43 more consultations over ten years than non-cancer controls. These findings align with prior research from the UK, Norway, and France showing sustained healthcare engagement following cancer diagnosis due to long-term treatment effects, multimorbidity, and surveillance needs [16,27,28,29]. The high consultation rates observed among Pakistani and Indian survivors may reflect greater multimorbidity burdens [26] or cultural factors influencing healthcare-seeking behaviours [30], though such explanations remain speculative given the absence of direct behavioural data. Conversely, Chinese survivors showed lower consultation rates, consistent with evidence of lower healthcare engagement in UK Chinese populations [31]. These results highlight associations rather than causal pathways between ethnicity and healthcare use; residual confounding from unmeasured social or cultural determinants may partially explain the observed differences.

A clear deprivation gradient was observed across all outcomes, with healthcare utilisation increasing in parallel with socioeconomic disadvantages. Similar gradients have been reported in other universal healthcare systems, including Norway and Denmark, suggesting that universal coverage alone does not eliminate inequities [8,9]. These associations likely reflect underlying social determinants of health such as multimorbidity, reduced preventive screening, and differences in health literacy and continuity of care. The findings underscore that greater healthcare use among deprived groups does not necessarily indicate better access or quality—it may instead signal unmet needs or delayed presentation.

Ethnic differences were prominent in hospital admission rates. Black Caribbean, Pakistani, and Bangladeshi survivors experienced the highest planned admission rates. For emergency admissions, only the Pakistani group retained a statistically significant elevation after adjustment, indicating possible differences in disease burden, care coordination, or barriers to timely care [32]. These patterns echo earlier UK findings of delayed presentation and higher emergency cancer diagnoses among ethnic minorities [4,28].

Higher healthcare utilisation among ethnic minorities and deprived groups may stem from multiple interrelated mechanisms: elevated multimorbidity, diagnostic delays, lower cancer awareness, and potential cultural or systemic barriers to accessing care [33,34]. Despite universal NHS coverage, such disparities persist, underscoring the need for tailored interventions that address both structural and behavioural determinants of health.

Importantly, these findings indicate associations between deprivation and healthcare use rather than direct causation. Higher utilisation among deprived groups may reflect both greater health needs and barriers to effective preventive care. Future studies incorporating longitudinal socioeconomic trajectories could help disentangle these effects.

From a policy perspective, integration between NHS primary and secondary care could reduce avoidable hospital use among deprived cancer survivors. Strengthening survivorship care planning in general practice—particularly in areas of high deprivation—may support earlier intervention, improve coordination, and lessen the need for emergency admissions. Community-level initiatives to improve cancer awareness and early symptom recognition among ethnic minorities could reduce emergency admissions and improve outcomes [35].

Strengths of this study include—it is the first study to investigate primary care consultations and hospital admissions (both planned and emergency) in cancer survivors, stratified by 28 different types of cancer, ethnicity, and the IMD. Previous studies have either examined fewer cancer types or focused on only one healthcare outcome, making this research a significant advancement due to its large population size and the ability to stratify results by multiple factors. The study found significant differences in healthcare use, particularly in primary care consultations and hospital admissions by ethnicity, and IMD. One of the key strengths of this analysis is the use of linked primary and secondary electronic health records (EHRs), which allows the inclusion of patients who have never been admitted to the hospital, offering a more accurate representation of hospital use compared to studies relying solely on hospital data. The use of primary care EHRs also provides a patient sample that better reflects the general population, as it includes individuals who may not have frequent hospital admissions. Additionally, the longitudinal nature of primary care records in England provides a richer dataset, enabling more detailed adjustments for factors such as age, sex, ethnicity, multimorbidity and deprivation. Matching cancer survivors with non-cancer controls from the same general practices helped minimise bias due to practice-level variations in service use.

However, several limitations warrant consideration. First, incomplete recording of long-term conditions (LTCs) in primary care may bias multimorbidity estimates, particularly for patients with infrequent consultations. Although cancer survivors typically have more primary care contacts, key variables such as BMI and smoking status showed similar levels of missingness across cancer and non-cancer groups, suggesting that increased consultations did not lead to increased capture of these variables. Second, the absence of detailed cancer-specific information—including cancer stage, recurrence status, treatment modality, treatment intensity, and treatment-related adverse events—limits interpretation of utilisation differences, as these factors strongly influence post-diagnosis care pathways. Third, the study lacked data on the cause of admission and psychosocial, behavioural, or patient-reported outcomes, all of which could mediate or modify patterns of healthcare use and contribute to residual confounding. Finally, although a complete case analysis was used due to the very large sample size and minimal missingness, some residual selection bias cannot be excluded; however, sensitivity analyses using multiple imputation demonstrated minimal differences in effect estimates, supporting the robustness of the findings.

Cancer survivors in England exhibit substantially higher primary-care consultations and hospital admissions than matched non-cancer controls. Disparities persist across ethnic and socioeconomic groups, with Pakistani, Bangladeshi, and Black Caribbean survivors showing particularly high planned admission rates and those from deprived areas experiencing the highest overall healthcare use.

## 5. Conclusions

To promote equity in survivorship care, NHS strategies should prioritise early diagnosis, improved management of comorbidities, and culturally adapted care pathways. Integration of primary care, oncology follow-up, and community health services could help reduce fragmented care and avoidable admissions. Routine inclusion of cancer stage, treatment modality, and patient-reported outcomes in linked datasets would enable more refined analysis of post-cancer healthcare needs. Future research should also explore qualitative perspectives on healthcare-seeking behaviours among minority and deprived survivors to inform culturally responsive interventions.

## Figures and Tables

**Table 1:** Key patient characteristics by cancer survivors and matched controls, n = 586,327.

Characteristic	Non-Cancer Patients	Cancer Survivors
Total number of patients (n, %)	415,975 (70.9%)	170,352 (29.05%)
Sex		
Male	182,386 (43.8%)	80,087 (47.0%)
Female	233,589 (56.2%)	90,265 (53.0%)
Age group		
18–30	2523 (0.6%)	867 (0.5%)
31–45	30,227 (7.3%)	10,245 (6.0%)
46–60	84,460 (20.3%)	28,632 (16.8%)
61–75	172,847 (41.6%)	61,998 (36.4%)
76–90	113,434 (27.3%)	58,864 (34.6%)
>90	12,484 (3.0%)	9746 (5.7%)
Ethnicity		
White	327,489 (78.7%)	156,236 (91.7%)
Mixed	1544 (0.4%)	651 (0.4%)
Asian or Asian British	10,368 (2.5%)	3341 (2.0%)
Black or Black British	4956 (1.2%)	2138 (1.3%)
Other	4301 (1.0%)	1701 (1.0%)
Unknown	15,022 (3.6%)	3224 (1.9%)
Missing	52,295 (12.6%)	3061 (1.8%)
IMD quintile		
1	104,784 (25.2%)	44,483 (26.1%)
2	95,027 (22.8%)	39,938 (23.4%)
3	86,632 (20.8%)	35,463 (20.8%)
4	72,682 (17.5%)	28,791 (16.9%)
5 (Most deprived)	56,850 (13.7%)	21,677 (12.7%)
Smoking status		
Non-smoker	220,523 (53.0%)	87,194 (51.2%)
Smoker	73,986 (17.8%)	28,071 (16.5%)
Former smoker	121,466 (29.2%)	55,087 (32.3%)
Alcohol drinking status		
Current drinker	224,266 (53.9%)	93,436 (54.8%)
Excess drinker	6995 (1.7%)	2518 (1.5%)
Former drinker	16,527 (4.0%)	7492 (4.4%)
Non-drinker	128,844 (31.0%)	51,682 (30.3%)
Missing	39,343 (9.5%)	15,224 (8.9%)
BMI		
Mean (SD)	26.8 (±5.2)	26.9 (±5.0)
Median (SD)	26.8 (±5.3)	26.9 (±5.1)
Male (Mean (SD))	27.3 (±4.6)	27.2 (±4.3)
Female (Mean (SD))	26.5 (±5.6)	26.6 (±5.6)
Underweight (BMI <18.5)	8226 (2.0%)	2451 (1.4%)
Normal (BMI 18.5–24.9)	160,919 (38.7%)	63,912 (37.5%)
Overweight (BMI 25.0–29.9)	153,902 (37.0%)	66,398 (39.0%)
Obese (BMI >30.0)	92,147 (22.2%)	37,304 (21.9%)
Missing	781 (0.2%)	287 (0.2%)
Death		
Mean age (SD)	79.0 (±10.7)	77.5 (±12.7)
Death above 75	35,337 (8.5%)	21,414 (12.6%)
Premature death (death below 75)	15,096 (3.6%)	11,997 (7.0%)
Multimorbidity		
>1 conditions	394,487 (94.8%)	163,155 (95.8%)
>2 conditions	336,763 (81.0%)	146,553 (86.0%)
>3 conditions	265,117 (63.7%)	125,729 (73.8%)
>4 conditions	202,332 (48.6%)	104,221 (61.2%)
>5 conditions	151,062 (36.3%)	83,857 (49.2%)

**Table 2:** Primary care consultations 2010–2020 by ethnicity, IMD and cancer status (highest-lowest IRR).

	Cancer Survivors			Non-Cancer Patients	
Ethnicity	Mean	Median (IQR)	Adjusted Incidence Rate (95% CI)	Mean	Median (IQR)
White	172	138 (67–238)	Reference	145	111(52–200)
Pakistani	188	134 (70–258)	1.16 (1.12–1.20)	170	128 (64–223)
Bangladeshi	157	114 (61–214)	1.14 (1.07–1.22)	167	125 (61.5–239)
Indian	182	138 (75–245)	1.11 (1.08–1.13)	166	130 (66–225)
Other Asian	151	117 (59–198)	0.96 (0.93–0.99)	150	115 (58–206)
Black Caribbean	167	133 (78–221)	0.95 (0.92–0.98)	146	114 (57–202)
Mixed	135	104 (48–183)	0.90 (0.87–0.94)	129	95 (43–173)
Black Others	132	104 (53.8–168)	0.90 (0.86–0.95)	134	100 (44–187)
Black African	137	105 (50–184)	0.87 (0.84–0.90)	129	98 (48–173)
Others	139	106 (49–191)	0.86 (0.84–0.88)	128	95 (44–171)
Chinese	128	101 (48–178)	0.77 (0.74–0.81)	123	93 (48–166)
IMD 1 (Least deprived)	161	129 (57–229)	Reference	114	80 (16–168)
IMD 2	159	127 (57–223)	1.00 (1.00–1.01)	115	82 (21–168)
IMD 3	165	132 (60–233)	1.07 (1.06–1.07)	123	87 (24–181)
IMD 4	169	134 (60–236)	1.13 (1.12–1.14)	128	92 (28–187)
IMD 5 (Most deprived)	175	136 (63–246)	1.23 (1.22–1.24)	136	98 (31–195)

**Table 3:** Planned hospital admission rates in cancer survivors by ethnicity and SES.

	Planned Admission		Emergency Admission	
	Person-Years (PY) of Follow-Up	Incidence per 1000 PY (95% CI)	Person-Years (PY) of Follow-Up	Incidence per 1000 PY (95% CI)
Ethnicity				
White	1,815,964,028	1.62 (1.62–1.63)	1,815,964,028	0.75 (0.75–0.75)
Bangladeshi	2,793,584	2.10 (2.05–2.16)	2,793,584	0.82 (0.78–0.85)
Black African	9,999,317	2.03 (2.01–2.06)	9,999,317	0.68 (0.66–0.70)
Black Caribbean	12,355,325	2.72 (2.69–2.75)	12,355,325	0.86 (0.84–0.88)
Black—Others	5,075,400	2.45 (2.40–2.49)	5,075,400	0.68 (0.65–0.70)
Chinese	4,750,558	1.55 (1.52–1.59)	4,750,558	0.46 (0.45–0.48)
Indian	23,732,648	2.01 (1.99–2.03)	23,732,648	0.74 (0.72–0.75)
Mixed	8,579,009	1.92 (1.89–1.95)	8,579,009	0.61 (0.59–0.63)
Other Asian	12,027,840	1.76 (1.73–1.79)	12,027,840	0.72 (0.70–0.73)
Other ethnicities	23,247,347	1.47 (1.46–1.49)	2,3247,347	0.54 (0.53–0.55)
Pakistani	9,566,149	2.79 (2.76–2.83)	9,566,149	1.01 (0.99–1.03)
IMD category				
IMD 1 (Least deprived)	571,615,907	1.31 (1.30–1.31)	571,615,907	0.50 (0.50–0.50)
IMD 2	291,857,066	1.63 (1.63–1.64)	291,857,066	0.93 (0.93–0.94)
IMD 3	381,169,715	1.54 (1.53–1.54)	381,169,715	0.76 (0.75–0.76)
IMD 4	512,246,208	1.43 (1.40–1.41)	512,246,208	0.59 (0.59–0.59)
IMD 5	461,054,398	1.47 (1.47–1.48)	461,054,398	0.65 (0.65–0.65)

## Data Availability

Restrictions apply to the availability of these data. Data were obtained from CPRD (study protocol 21_000345) and are available at https://www.cprd.com/data-access with the permission of CPRD’s Independent Scientific Advisory Committee for the Medicines and Healthcare products Regulatory Agency.
